# Chemical Treatments for Insect Cell Differentiation: The Effects of 20-Hydroxyecdysone and Veratridine on Cultured *Spodoptera frugiperda* (*Sf*21) Insect Cell Ultrastructure

**DOI:** 10.3390/insects13010032

**Published:** 2021-12-28

**Authors:** Lacey J. Jenson, James J. Becnel, Jeffrey R. Bloomquist

**Affiliations:** 1 Department of Entomology, Emerging Pathogens Institute, University of Florida, Gainesville, FL 32601, USA; lcole@bedoukian.com; 2 Center for Medical, Agricultural, and Veterinary Entomology, USDA-ARS, Gainesville, FL 32601, USA; james.becnel@ars.usda.gov

**Keywords:** cellular toxicity, mitochondrial granules, transmission electron microscopy

## Abstract

**Simple Summary:**

Cultured insect *Sf*21 cells treated with the hormone 20-hydroxyecdysone grow long processes and resemble neurons. They also make physical contact with one another and appear to have the potential to form synapses, areas in which nerve cells are in close contact and communicate with one another electrically or by the release of chemical transmitters. This study uses electron microscopy to look for structural evidence of synapses in 20-hydroxyexdysone treated *Sf*21 cell cultures. Unfortunately, no evidence of synaptic structures were observed, suggesting that other factors are required for the formation of functional synapses in these cultures.

**Abstract:**

Previous studies have shown that insect cell cultures stop dividing, form clumps, and can be induced to grow processes reminiscent of axons, when the culture medium is supplemented with 20-hydroxyecdysone, insulin, or an agent that mimics their action, such as the ecdysone agonist, methoxyfenozide. Those cell growing processes resemble nerve cells, and the present study evaluates the ultrastructure of these cultures by transmission electron microscopy. *Sf*21 cells treated with 20-hydroxyecdysone (with or without veratridine amendment) and subjected to ultrastructural analysis had a similar somatic appearance to control cells, with slight changes in organelles and organization, such as a greater number of cytoplasmic vacuoles and mitochondrial granules. Finger-like projections were observed between control and treated cells. However, no structural markers of synaptic contacts (e.g., vesicles or synaptic thickenings) were observed in controls, 20-hydroxyecdysone, or 20-hydroxyecdysone + veratridine treated cells. It is concluded that additional agents would be required to induce functional synaptogenesis in *Sf*21 cells.

## 1. Introduction

The active metabolite of ecdysone is 20-hydroxyecdysone (20-HE), and many insect species use it as a regulator of molting and other processes, including the control of neuronal ion channel expression during development [1]. In an early study with Kc-H cells, 20-HE caused the mostly spherical cells to stop dividing, clump together, and grow long processes; effects that were maximal at about 10 nM 20-HE [2]. Similar effects were documented in subsequent studies with lepidopteran cell lines [3,4,5,6,7]. In the results reported by Jenson et al. [7], the proportion of cells with processes showed a biphasic concentration curve, saturating at about 0.1–1 µM 20-HE within 2 days and increasing again at higher concentrations of up to 50 µM. At 100 µM, the morphological effects of 20-HE were reduced, perhaps due to cellular toxicity. Similar effects on cell growth were observed in *Sf*9 (*Spodoptera frugiperda*) cells treated with the insecticide methoxyfenozide, which mimics the actions of 20-HE and blocked cell proliferation and initiated cell arrest [8].

Subsequent studies demonstrated the expression of neuron-like pharmacological properties in 20-HE treated insect cell lines. On Sua-1b (*Anopheles gambiae*) cells, 20-HE stimulated the expression of neonicotinoid (imidacloprid)-induced calcium fluorescence responses [9], as well as Kv2-type potassium channel currents in patch-clamped Sua-1b cells [10]. The effects of 20-HE treatment in *Sf*21cells were sensitive to blockage by caffeine [7], as well as cobalt ion [11], which blocks plasma membrane calcium channels [12]. In addition, while 1 µM veratridine (VTD), a lipophilic plant toxin that activates voltage-sensitive sodium channels [13], was inactive on *Sf*21 cells by itself, it synergized the morphological effects of 20-HE and this action was reversed by the specific sodium channel blocker, tetrodotoxin [11]. A more recent study showed that 2 μg/mL 20-HE can induce electrical activity in the RML12 mosquito cell line grown on microelectrode arrays, and the cells responded to incubation with GABAzine, nicotine, permethrin, and temephos [14].

Ultrastructure is a powerful tool to examine the structural, morphological, and functional development of a wide variety of tissues. Baculovirus expression studies, as well as virus identification and replication, have been widely documented in insect cell lines using transmission electron microscopy [15,16]. Additionally, Marks and Ward [17] reported cuticular particulates in the UMBGE-2 *Blattella germanica* cell line using ultrastructural techniques. However, no ultrastructural information is available following 20-HE application to insect cell lines, including those documenting the stimulation of cell process growth mentioned above. In the present study, we are interested in whether 20-HE treatment can induce the presence of ultrastructural markers of synaptogenesis in *Sf*21 cells, the results of which are presented here.

## 2. Materials and Methods

### 2.1. Chemicals and Cell Culture

The chemicals used in treatment flasks were DMSO (vehicle), 20-HE, and VTD, and all were obtained from Sigma-Aldrich (St. Louis, MO, USA). The *Sf*21 insect cell line was obtained from Invitrogen (Carlsbad, CA, USA). The cells were maintained in a log phase culture in tissue culture flasks (BD Falcon, Fisher Scientific, Suwanee, GA, USA) treated with *Trichoplusia ni* Medium-Formulation Hink (TNM-FH) from Sigma-Aldrich. Insect media was supplemented with 10% fetal bovine serum (Sigma-Aldrich) and 100 U/mL penicillin and streptomycin (Sigma-Aldrich). Cells were maintained at 27 °C in a non-humidified incubator (Amerex Instruments, Inc., Lafayette, CA, USA).

### 2.2. Differentiation of Cells

Once a confluent monolayer of cells formed in the culture flask, the cells were sloughed off and transferred to another sterile flask. The quantity of cells transferred was determined by the area of the growth surface to give a 1:5 dilution (cells/cm^2^), as in a normal passage. Fresh growth medium was added and the cells were allowed to attach for 30 min in the incubator. The medium was then removed and fresh medium containing either 0.1% DMSO (control), 20-HE in DMSO (final concentration was 20 μg/mL or 42 µM), or 20-HE + VTD in DMSO (final concentration 1 µM VTD), was added to the culture. VTD was used at this concentration because 1 µm was inactive alone, but enhanced the effects of 20-HE on *Sf*21 cells, as mentioned above [7]. Cell cultures were processed when maximal differentiation occurred in *Sf*21 cells after exposure to agents, 48–72 h [7].

### 2.3. Tissue Preparation and Electron Microscopy

Control and hormonally-treated cells were fixed in a 2.5% (*v*/*v*) glutaraldehyde solution buffered in 100 mM Na cacodylate (pH 7.4) for 2 h at 4 °C, post-fixed in aqueous 1% (*w*/*v*) OsO_4_ (pH 7.4) for 2 h at room temperature, dehydrated through a graded ethanol and acetone series, and embedded in Epon 812-Araldite (Fluka, Switzerland). Thin sections (60–100 nm) were stained with 2% (*w*/*v*) uranyl acetate in 50% ethanol, followed by Reynolds’s lead citrate [18], and examined in a transmission electron microscope (TEM) at an accelerating voltage of 75 kV (Hitachi High-Technologies Europe GmbH, Krefeld, Germany).

### 2.4. Statistical Analyses

Transmission electron microscope images were analyzed by determining the calculated mean and standard error of the mean (SEM) for mitochondria, vacuoles, and mitochondrial granules from cell photographs of each chemical treatment, either alone or in combination using GraphPad Prism^TM^ (GraphPad Software, San Diego, CA, USA). Comparisons were then made between treatment groups by using a one-way analysis of variance procedure (ANOVA), followed by a multiple comparison test using the same software.

## 3. Results

**Figure 1:**
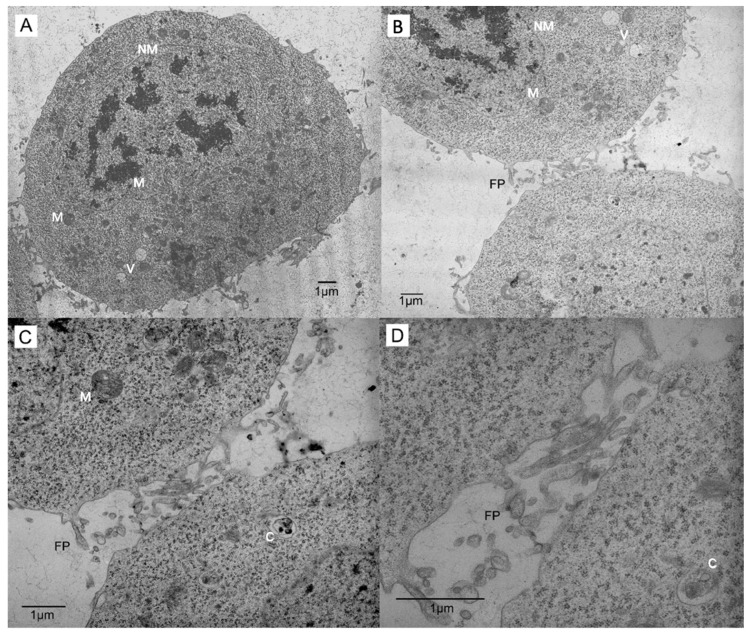
Increasing magnifications of *Sf*21 control cells demonstrating cell-to-cell connections via finger-like projections of the plasmalemma, which can be remnants of cell division. (**A**) Control cells are round and show intact nuclear membrane (NM), mitochondria (M), and vacuoles (V). (**B**–**D**) Examples of finger-like projections (FP) observed between cells, along with circular vacuoles (V) and cytolysosomes (C).

The morphology and ultrastructural analysis of control cells are shown in Figure 1.

Control *Sf*21 cells are generally spherical (Figure 1A); however, other shapes are present, and only the occasional elongated cell with a short process appeared in the monoculture, as we described previously [7]. The ultrastructure of control *Sf*21 cells showed basic cellular components typical of any vertebrate or invertebrate cells (Figure 1A–C). Finger-like projections between closely-apposed cells were sometimes observed and were present more often in control cells (Figure 1D).

**Figure 2:**
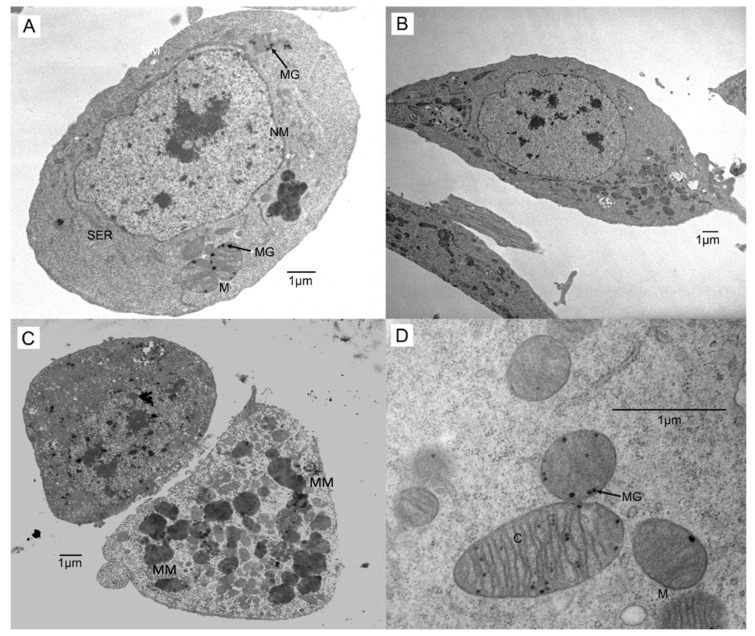
Electron micrographs illustrating the ultrastructure of treated cells. (**A**) A 20-HE treated cell with no morphological differentiation. The cell structure shows circular mitochondria (M) with densely stained mitochondrial granules (MG), smooth endoplasmic reticulum (SER), and a fully intact nuclear membrane (NM). (**B**) A 20-HE treated elongated cell showing morphology similar to Figure 3B. (**C**) Mitochondrial malformations (MM) in a cell treated with 20-HE and VTD. (**D**) Mitochondria (M) from a cell treated with 20-HE having numerous densely stained granules (MG) within the mitochondria and cristae (C).

In terms of the ultrastructural effects of 20-HE and VTD treatments, mitochondrial size varied more in treated cells than control, with most examples twice the size of control mitochondria, regardless of whether treated cells were round (Figure 2A) or elongated (Figure 2B). The mitochondria often occurred in clusters within the cytoplasm of control and treated cells, but the aggregates were normally larger in size for cells treated with 20-HE or 20-HE/VTD in combination (Figure 2C). Many of the mitochondria in treated cell aggregates were deformed (Figure 2C) and had numerous densely stained bodies or granules within them, some of which were also observed in controls. Overall, organelle organization within the cell appeared to possess increased variability in treated cells versus control cells.

**Figure 3:**
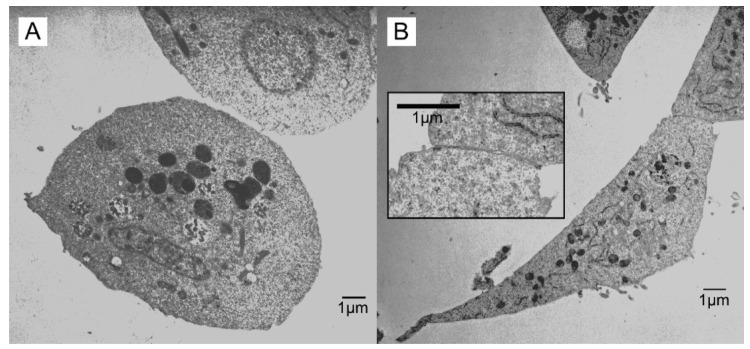
Cell-to-cell contacts in 20-HE treated *Sf*21 cell cultures. (**A**) A cell showing only a single finger-like projection with no additional membrane or closely associated cytoplasmic elaborations. (**B**) Close apposition between elongated cells. Inset is at greater magnification and shows no syn-aptic or septate junction-like morphology between the two cells.

Cell-to-cell contacts arising after 20-HE treatment are shown in Figure 3. When cell-to-cell contact occurred, it was manifested in two different ways. The first was the finger-like projections (Figure 3A), similar to those observed in controls. The other was a region of closely apposed membranes between two elongated cells (Figure 3B). We observed no examples for which this type of close contact showed any ultrastructural features of chemical synapses, such as pre- and postsynaptic thickenings and synaptic vesicles [19].

**Figure 4:**
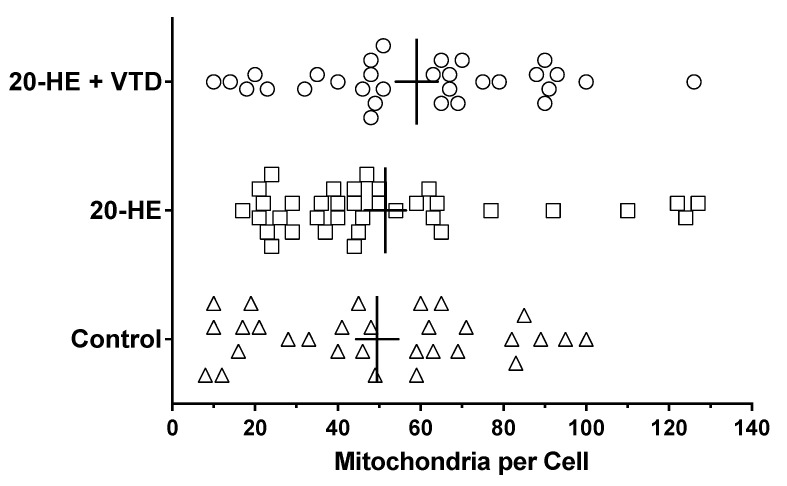
Number of mitochondria per *Sf*21 cell. Treatments were 0.1% DMSO (control), 42 μM 20-HE, and 42 μM 20-HE + 1 μM VTD. The vertical line for each group of symbols demarcates the mean with the SEM shown as a horizontal bisecting line. Statistical analysis was performed using one-way ANOVA, but no post-test due to a lack of statistical significance (*p* = 0.386). Sample size was at least *n* = 30 cells/treatment.

**Figure 5:**
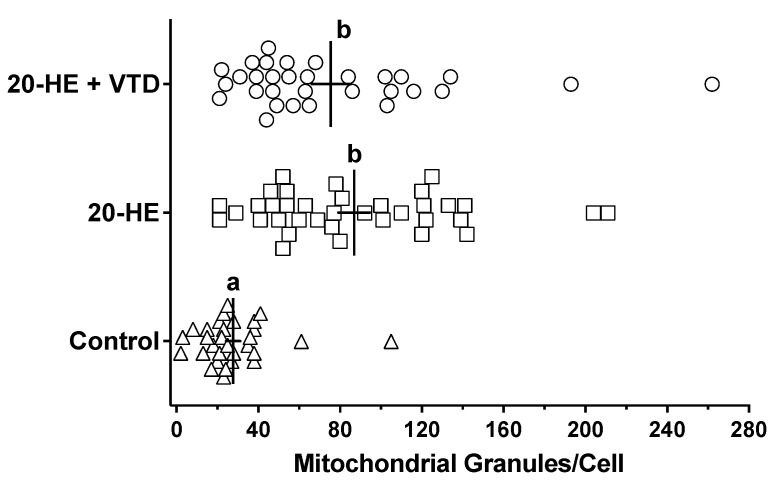
Distribution of mitochondrial granules per cell in *Sf*21 cell cultures. Treatments were 0.1% DMSO (control), 42 μM 20-HE, and 42 μM 20-HE + 1 μM VTD. The vertical line for each group of symbols demarcates the mean with the SEM shown as a horizontal bisecting line. Statistical analysis used one-way ANOVA and a Tukey’s multiple comparison post-test. Bars not labeled by the same lower-case letter, indicate the statistical significance across treatments (*p* < 0.0001). Sample size was at least *n* = 30 cells/treatment.

Ultrastructural quantitative analysis included measures of mitochondrial numbers and their associated granules. For all three treatment groups, the number of mitochondria/cell was variable and ranged from a low of about 10/cell to >100/cell (Figure 4). The average was about 50–60/cell and did not differ across all treatment groups (ANOVA, *p* = 0.386, n > 30). The quantitation of mitochondrial granules found they were about 3-fold more numerous in mitochondria from the 20-HE and 20-HE + VTD treatment groups than in controls (*p* < 0.05), and varied more in the number of granules/cell (Figure 5). The 20-HE- and 20-HE + VTD-treated groups were not significantly different from each other.

## 4. Discussion

Ultrastructural analysis showed control cells with finger-like projections, which were more prevalent in control cultures (Figure 1). The cell surface uses extensions for the conduct of many different cellular processes, including cell migration, phagocytosis, neurite outgrowth, cell-to-cell junction formation, and cell signaling [20]. These projections may be acting as one of many types of membrane extension; however, a formation similar to microvilli, which are used to help the cell to increase surface area and for nutrient and water uptake [20], seems likely. Alternatively, they can be a remnant of cell division, since they were seen more commonly in control cultures that were still actively dividing. Additional research will be needed to identify the specific functions for these processes in 20-HE treated cultures.

The development of cell–cell contacts in 20-HE-treated insect cell cultures were observed by Lynn and Oberlander [3], including some similar in morphology to that shown in Figure 3. Although we observed close intercellular contacts in the present study, no evidence of chemical synaptic structures (e.g., vesicles or pre- and postsynaptic thickenings) was observed. Thus, we conclude that additional differentiating factors are required to induce synaptogenesis and that any induced expression of ion channels or receptors occurs in the absence of physical synapses. The expression of ion channels and extrasynaptic receptors is commonly observed in electrophysiological studies of acutely isolated neurons. Electrical excitability, as well as receptors for a variety of neurotransmitters, such as acetylcholine, octopamine, serotonin, and γ-aminobutyric acid, were observed on freshly dissociated locust somata [21], even though they lacked any obvious synaptic structures.

Although the average number of mitochondria/cell was not changed, variations in morphology, such as dumbbell shaped mitochondria, were demonstrated in hormone-treated *Sf*21 cells when compared to controls (Figure 2). Normann and Samaranayaka-Ramasamy [22] also observed dumbbell shaped mitochondria in lindane-treated cell aggregates, as were observed in our study. This observation was ascribed to various stages of mitochondrial fission taking place within the cells and represents the division of the mitochondria and not the fusion. Thus, this effect can be a general response to sublethal toxic insult or cell stress, including, in the present study, the effects of high concentrations of the insect hormone, 20-HE.

Another significant finding in the ultrastructure of treated Sf21 cells is the appearance of mitochondrial granules. Mitochondrial granules are electron-dense areas that appear as dark spots inside cellular mitochondria [23]. These granules have been found in plant material, and a variety of developing and adult mammalian tissues, including those undergoing pathological changes [23], as well as in insects [22]. Many hypotheses have been put forward regarding the nature and physiological functions of these dense granules, which were first observed in the 1950s and are thought to be precipitations of calcium ions and phospholipids, among other constituents, that appear to create contact sites between inner and outer mitochondrial membranes for efficient enzyme function [23]. In the studies of Normann and Samaranayaka-Ramasamy [22], it was suggested that in lindane-treated corpora cardiac cells, there occurred a greater uptake of calcium by the mitochondria from cell stress. It would be interesting in future studies, to correlate the findings presented in our study with the ultrastructure of cells differentiated by lower concentrations of 20-HE, as well as other treatments (e.g., insulin or caffeine), different cell lines, or various combinations of differentiating factors.

## 5. Conclusions

Ultrastructural analysis was performed on closely apposed cells, in order to document any membrane structures reminiscent of chemical synapses. Unfortunately, no structural markers of chemical synapses were observed in the present study (Figure 3), indicating that additional differentiating factors are required to induce synaptogenesis in these cultures. Further, any hormone-induced expression of ion channels or neurotransmitter receptors would be extrasynaptic in nature. Finally, the functional impact on cell death and differentiation for the mitochondrial granules observed in 20-HE treated cells remain to be determined.

## Data Availability

The data presented in this study are available on request from the corresponding author. The data are not publicly available due to intellectual property considerations.
